# Reductive Methylation: An Alternative to Lysine → Arginine Mutagenesis

**DOI:** 10.1002/psc.70110

**Published:** 2026-06-16

**Authors:** Oscar J. Molina, Clair S. Gutierrez, Jinyi Yang, Evans C. Wralstad, Ronald T. Raines

**Affiliations:** ^1^ Department of Biology Massachusetts Institute of Technology Cambridge Massachusetts USA; ^2^ Department of Chemistry Massachusetts Institute of Technology Cambridge Massachusetts USA

**Keywords:** bioconjugation, posttranslational modification, protein stability, protein–protein interaction, ribonuclease, targeted protein degradation

## Abstract

Modification of lysine residues is a common strategy in protein engineering, whether to prevent posttranslational modifications, control bioconjugation, or improve crystallization. The standard genetic approach—replacement with arginine by site‐directed mutagenesis—preserves positive charge but alters other physicochemical attributes and cannot address the N‐terminal amino group. Here, we characterize reductive methylation as a chemical alternative. This reaction converts every primary amino group to a dimethylamino group rapidly under mild aqueous conditions. Using human ribonuclease 1 and a cytotoxic variant engineered to evade the endogenous ribonuclease inhibitor as model systems, we assess the effects of complete dimethylation on thermostability, enzymatic catalysis, protein–protein interaction, compatibility with bioconjugation, cellular uptake, and intracellular persistence. Dimethylation preserves thermostability and a protein–protein interaction. Enzymatic catalysis, in contrast, is reduced by 10^2^‐ to 10^3^‐fold, consistent with the role of catalytic lysine residues. Dimethylation is fully compatible with bioconjugation chemistry. Dimethylated and unmodified ribonucleases show comparable uptake and persistence in human cells. These findings establish reductive methylation as a practical and conservative strategy for lysine modification in protein and peptide engineering and support its use in applications such as biological proteolysis‐targeting chimeras (bioPROTACs).

## Graphical abstract

Replacing lysine with arginine, the standard genetic approach to eliminate amine reactivity, alters physicochemical properties and leaves the N terminus unprotected. Here, reductive methylation is characterized as a conservative chemical alternative. Complete dimethylation of human ribonucleases preserves thermostability, protein–protein interaction, cellular uptake, and intracellular persistence, while predictably diminishing enzymatic catalysis. Fully compatible with bioconjugation, this rapid modification is a practical strategy for protein and peptide engineering and developing intracellular biologics such as bioPROTACs.

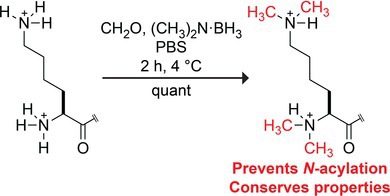

## 1 Introduction

Among the canonical amino acids, none rivals lysine in the chemical diversity of its posttranslational modifications [1, 2, 3, 4, 5]. The *ε*‐amino group serves as the attachment point for methyl [6, 7, 8], acetyl, and ubiquitin‐like modifiers, while its protonated form mediates the Coulombic interactions and hydrogen bonds that drive protein stability, substrate binding, and enzymatic catalysis [9, 10]. This versatility makes lysine a frequent target of protein engineering: researchers modify lysine residues to prevent ubiquitination and extend protein half‐life [11, 12, 13], to improve crystallization [14, 15], or to dissect contributions to function [16, 17]. As the field of targeted protein degradation matures and protein‐based degraders (bioPROTACs) move toward intracellular applications [18, 19, 20, 21, 22, 23, 24], the ability to modify lysine residues in a controlled, well‐characterized manner has taken on greater practical importance.

**FIGURE 1:**
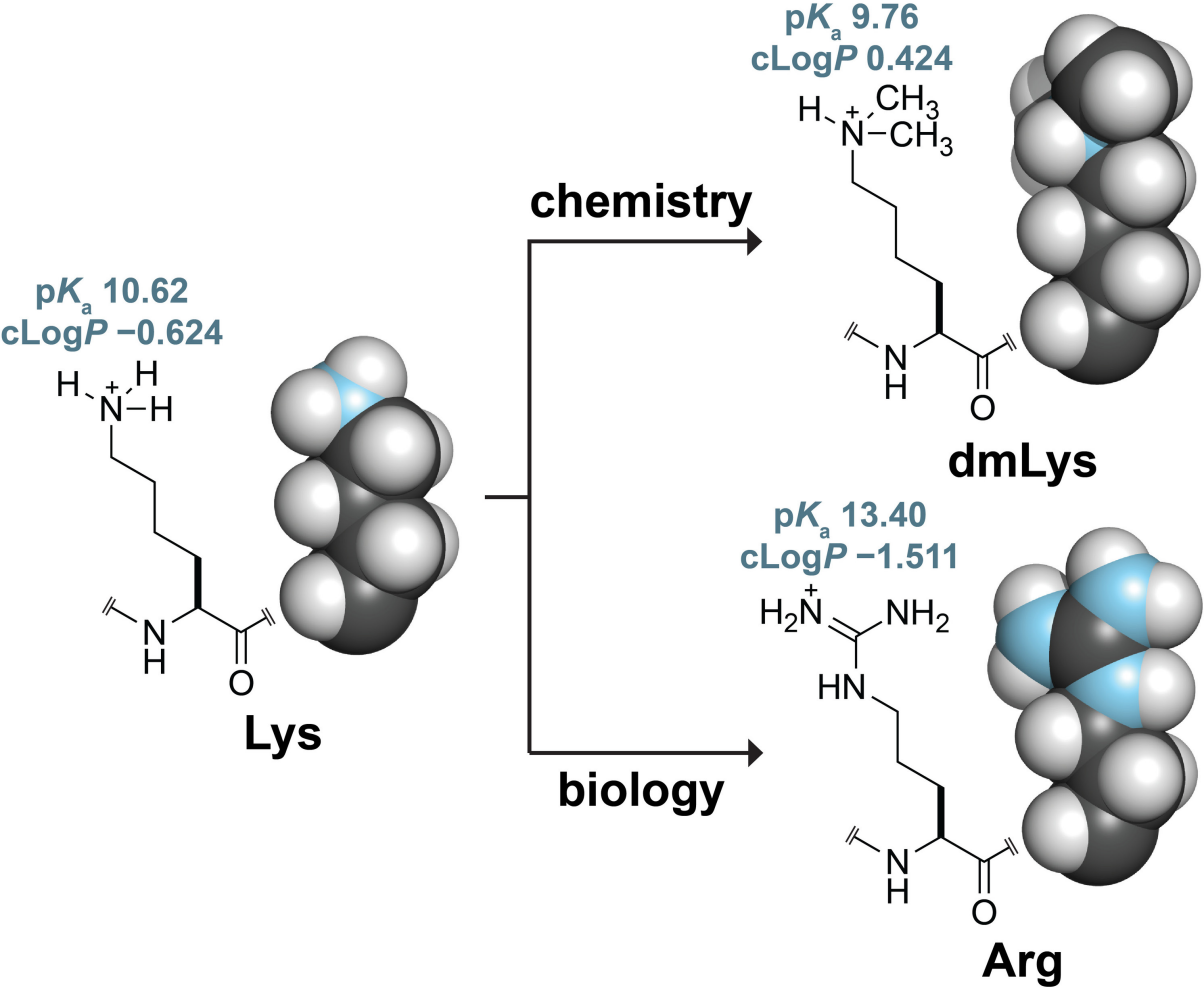
Conservative chemical and biological alterations to an l‐lysine residue (Lys). Lys can be converted to a *N* ^ε^,*N* ^ε^‐dimethyl‐l‐lysine residue (dmLys) by reductive methylation. Lys can be converted to an l‐arginine residue (Arg) by site‐directed mutagenesis. p*K*_a_ and cLog*p* values are for the conjugate acids of methylamine (Lys) [28], trimethylamine (dmLys) [28], and methylguanidine (Arg) [29]. Side chains are depicted as space‐filling models.

The standard genetic approach to eliminating the reactivity of a lysine residue is its replacement with arginine via site‐directed mutagenesis [25, 26, 27]. Arginine preserves a positive charge at physiological pH and approximates the side‐chain length of lysine. Nonetheless, the two residues differ in important respects (Figure 1). The guanidinium group of arginine has a p*K*_a_ approximately 2.8 units higher than the ammonium group of lysine, rendering it essentially permanently protonated under biological conditions [28, 29]. The planar, Y‐shaped guanidinium group presents five hydrogen‐bond donors in a geometry distinct from that of the tetrahedral ammonium group of lysine, which has three donors, and its larger van der Waals surface can perturb packing at protein–protein interfaces. Moreover, substitutions can require multiple rounds of mutagenesis, and the approach cannot address the N‐terminal *α*‐amino group, which is itself a substrate for ubiquitination [11, 30] and other modifications.

Reductive methylation offers a chemical alternative that, in principle, is more conservative (Figure 1). Indeed, before the advent of routine site‐directed mutagenesis, reductive methylation was the foundational technique for blocking ubiquitination. Reductively methylated substrates and “chain‐terminating” reductively methylated ubiquitin were instrumental in deciphering the ubiquitin‐proteasome pathway [31, 32, 33, 34]. The reaction can convert *every* primary amino group, both the *ε*‐amino groups of lysine side chains and the *α*‐amino group of the N terminus, to a dimethylamino group through imine formation with formaldehyde, followed by reduction with a mild hydride donor [14, 15]. Only two CH_2_ groups (six atoms) are added per site. The resulting dimethylammonium group retains a positive charge at physiological pH, with a p*K*_a_ shift of only −0.8 units relative to the parent ammonium group (Figure 1), and it can no longer serve as a substrate for ubiquitin ligases. The reductive methylation reaction proceeds to completion under mild aqueous conditions (4°C, pH 7.5) within hours and has been used as a routine rescue strategy for protein crystallization [15]. Despite its simplicity, reductive methylation has not been characterized systematically, an omission that leaves protein engineers and peptide chemists without quantitative guidance on how the modification affects function.

Here, we use ribonucleases as a model system to fill that gap. Ribonuclease (RNase) 1, a human homolog of bovine pancreatic ribonuclease (RNase A), and QBI‐139, a variant that manifests cytotoxic ribonucleolytic activity [35, 36, 37, 38], together present a rich set of testable properties: both proteins have eight lysine residues and an N terminus available for modification; three lysine residues (Lys7, Lys41, and Lys66) are intimately involved in substrate binding and turnover [17, 39, 40]; and QBI‐139 engages RI in a protein–protein interaction [37, 38]. We characterize the effects of complete dimethylation on thermostability, enzymatic catalysis, affinity for RI, compatibility with orthogonal bioconjugation chemistry, cellular uptake, and intracellular persistence. Our results demonstrate that reductive methylation is conservative for many protein properties with the notable exception of catalysis, where the modification is consequential.

## 2 Materials and Methods

### 2.1 Conditions

All procedures were performed at ambient temperature (∼22°C) and pressure (1.0 atm) unless indicated otherwise.

### 2.2 Materials

Commercially available reagents and solvents were reagent grade or better from Sigma–Aldrich (St. Louis, MO), unless specified otherwise, and were used directly without further purification. RNase A (≥70 Kunitz units mg^−1^) was product #R6513, and BL21(DE3) *Escherichia coli* cells were product #69450‐3 from Sigma‐Aldrich.

A 3.5 kDa MWCO dialysis tubing was from Spectrum Labs (Rancho Dominguez, CA). Amicon 15 mL 10 kDa MWCO centrifugal filters were from Thermo Fisher Scientific (Waltham, MA). Zeba spin desalting columns (0.5 mL, 7 kDa MWCO) were from Thermo Fisher Scientific (product #89882). Clear tissue culture‐treated 96‐well plates with flat‐bottomed wells were from Greiner (Kremsmünster, Austria) (product #655160). CellTiter 96 AQueous One Solution Cell Proliferation Assay was from Promega (Madison, WI) (product #G3580). The 0.5 M sodium phosphate buffer, pH 7.0, was from Thermo Fisher Scientific (product #J63791.AK).

### 2.3 Instruments

*E. coli* cells were lysed with a benchtop cell disruptor from Constant Systems (Daventry, UK). FPLC was performed with an ÅKTA pure FPLC system from Cytiva (Wilmington, DE). Protein concentrations were determined using a DS‐11 UV–vis spectrophotometer from DeNovix (Wilmington, DE) and validated using a BCA assay kit (Thermo Fisher Scientific, product #675801). Absorbance measurements were made with a Spark plate reader from Tecan (Männedorf, Switzerland). Protein ESI mass spectrometry was performed with a 6350 Accurate‐Mass Q‐TOF LC/MS instrument from Agilent Technologies (Santa Clara, CA) equipped with a PLRP‐S column (1000 Å pore size, 5‐μm particle size, 50 mm length × 2.1 mm ID). Q‐TOF LC/MS measurements used a linear gradient (5%–95% v/v) of ACN (0.1% v/v formic acid) in water (0.1% v/v formic acid) over 15 min. Peptides were synthesized with a Liberty Blue automated microwave peptide synthesizer from CEM (Matthews, NC). Peptide mass was determined with a MALDI‐TOF microflex LRF instrument from Bruker (Billerica, MA).

Data were plotted and analyzed with Prism 11.0.0 from GraphPad Software (San Diego, CA). Values of cLog*P* were calculated with ChemDraw 20.1.0.112 from PerkinElmer (Shelton, CT).

### 2.4 Production and Purification of Ribonucleases

QBI‐139 was a gift from Dr. L. E. Strong (Quintessence Biosciences). M‐DDDDK‐RNase 1 and A19C/G88R RNase A were produced in *E. coli* without a signal peptide but with an N‐terminal methionine residue essentially as described previously [41, 42, 43]. M‐DDDDK‐RNase 1 was used to make authentic RNase 1 (i.e., without an N‐terminal methionine residue) after enterokinase cleavage [43]. A19C/G88R RNase A is an RI‐evasive variant with Cys19 installed for conjugation [41].

In C4R/P19C/C118V QBI‐139, the fifth disulfide bond in QBI‐139 was reverted to its wild‐type residues, and Cys19 was installed for conjugation. Expression plasmids were transformed into BL21(DE3) *E. coli* cells. A 50 mL starter culture was inoculated from a single colony and grown overnight at 37°C with constant shaking at 250 rpm in terrific broth (TB) containing ampicillin (200 μg mL^−1^). 1‐L cultures were initiated from the starter culture at an OD_600 nm_ = 0.05 and grown at 37°C in TB containing ampicillin (200 μg mL^−1^) with constant shaking at 250 rpm. Gene expression was induced with a final concentration of 1.0 mM isopropyl‐β‐d‐1‐thiogalactopyranoside (IPTG) when cultures reached OD_600 nm_ = 1.8 and were grown for an additional 3 h at 37°C with constant shaking at 250 rpm. Cells were pelleted by centrifugation at 6000 *g* for 15 min at 4°C, and cell pellets were stored at −80°C until resuspension and lysis.

To reduce any mixed disulfides with Cys19, tris(2‐carboxyethyl)phosphine (TCEP) was added at a fivefold molar excess, and the resulting solution was incubated at 4°C for 1 h. The solution was then loaded onto a HiTrap SP cation‐exchange column in 50 mM sodium acetate buffer, pH 5.0, and C4R/P19C/C118V QBI‐139 was eluted with 1.0 M NaCl. Fractions containing C4R/P19C/C118V QBI‐139 were pooled, and the pH was adjusted to 8.0 with 1.0 M Tris–HCl buffer, pH 8.0. DTNB was added at a fivefold excess from a 5 mM stock solution in 50 mM Tris–HCl buffer, pH 8.0, containing EDTA (10 mM) and NaCl (50 mM). Upon incubation at 4°C for 10 min, the solution turned yellow. The pH was adjusted back to 5.0 with 3.0 M sodium acetate buffer, and the mixture was incubated at 4°C overnight. The resulting sample was further purified using a HiTrap SP cation‐exchange column with a linear gradient of NaCl (0.0–1.0 M) in 50 mM sodium acetate buffer, pH 5.0, over 20 column volumes. Fractions containing C4R/P19C/C118V QBI‐139 were pooled, concentrated, and exchanged into Tris–HCl buffer, pH 7.2, containing 50 mM NaCl using an Amicon 15 mL 10‐kDa MWCO spin concentrator. Protein identity was confirmed with SDS–PAGE and Q‐TOF mass spectrometry. Aliquots were flash‐frozen in N_2_(l) and stored at −80°C.

### 2.5 Production and Purification of Ribonuclease Inhibitor

Recombinant human RI was produced in *E. coli* and purified essentially as described previously [44]. Purified RI was dialyzed into 25 mM HEPES–NaOH buffer, pH 7.5, containing NaCl (150 mM), DTT (10 mM), and glycerol (10% v/v), flash‐frozen in N_2_(l), and stored at −80°C.

### 2.6 Reductive Methylation

Reductive methylation of RNase 1 and QBI‐139 was performed as described previously [15] with modifications. Purified protein was diluted to 1–2 mg mL^−1^ in 50 mM sodium phosphate buffer, pH 7.5. Borane·dimethylamine complex was added from a 1.0 M stock solution at a 10‐fold excess over the number of protein amino groups. Then, formaldehyde was added from a 1.0 M stock in a 20‐fold excess relative to the number of amino groups on the protein. The reaction mixture was incubated for ≥2 h at 4°C. The procedure was repeated to ensure complete labeling. After the second incubation, free glycine was added to a final concentration of 100 mM to sequester the formaldehyde, and the reaction mixture was incubated for 2 h at 4°C. Dimethylated proteins were exchanged into DPBS without Ca^2+^/Mg^2+^ using an Amicon 0.5 mL 10‐kDa MWCO spin concentrator for cytotoxicity assays or into 50 mM Tris–HCl buffer, pH 7.5, containing NaCl (50 mM) for other assays. Reductively methylated proteins are designated with the prefix “dm”.

### 2.7 Protein Thermostability Assay

The thermostabilities of RNase 1, QBI‐139, and their dimethylated variants were assessed in 100 mM Tris–HCl buffer, pH 7.5, containing NaCl (100 mM) using differential scanning fluorimetry as described previously [45].

### 2.8 Enzymatic Activity Assay

Ribonucleolytic activity was measured in 100 mM Tris–HCl buffer, pH 7.5, containing NaCl (100 mM) using a fluorogenic tetranucleotide substrate, 6‐FAM–dArU (dA)_2_–6‐TAMRA, as described previously [45].

### 2.9 Preparation of DEF–G88R RNase A

DEF–G88R RNase A was prepared essentially as described previously [41]. Briefly, NTB‐capped A19C/G88R RNase A was deprotected with tris(2‐carboxyethyl) phosphine, and the nascent cysteine was *S*‐alkylated with the pH‐sensitive fluorophore, 2′,7′‐diethylfluorescein‐5‐iodoacetamide (DEFIA), which was a gift from Dr. L. D. Lavis (University of Wisconsin–Madison).

### 2.10 Affinity for Ribonuclease Inhibitor

The affinity of QBI‐139 and dmQBI‐139 for RI was measured with a competition assay, as described previously [41]. In this assay, the fluorescence of RI‐bound DEF–G88R RNase A in PBS containing BSA (0.1 mg mL^−1^) and DTT (10 mM) increases upon dissociation driven by competition with QBI‐139 or dmQBI‐139.

### 2.11 Human Cell Culture

K‐562 human myeloid leukemia cells and HAP1 human near‐haploid leukemia cells were obtained from the Robert A. Swanson (1969) Biotechnology Center at the Koch Institute for Integrative Cancer Research at MIT. HEK 293AAV LgBiT human embryonic kidney cells were a gift from Prof. G. M. Church (Harvard Medical School). K‐562 cells were grown in RPMI‐1640 from Thermo Fisher Scientific (product #11875093). HAP1 cells were grown in IMDM from Thermo Fisher Scientific (product #12440053). HEK 293AAV LgBiT cells were grown in DMEM from Thermo Fisher Scientific (product #11995081). RPMI‐1640, IMDM, and DMEM were supplemented with fetal bovine serum from Corning (product #45001‐108) and a solution of penicillin (10^4^ units mL^−1^)–streptomycin (10^4^ μg mL^−1^) from Thermo Fisher Scientific (product #15140122) to make complete media. Cells were cultured in an incubator maintained at 37°C and humidified to 5% v/v CO_2_(g). Cells were collected with TrypLE Express (Thermo Fisher Scientific, product #12605010) during passaging. All experiments were performed with cells that had <20 passages and tested negative for mycoplasma using the MycoAlertTM PLUS Assay from Lonza Biosciences (product #LT07‐710) at the Koch Institute for Integrative Cancer Research at MIT.

### 2.12 Human Cell Viability Assay

Cell viability in the presence of QBI‐139 and dmQBI‐139 was measured using the CellTiter 96 AQueous One Solution Cell Proliferation Assay. The metabolic activity of viable cells was measured through the conversion of 3‐(4,5‐dimethylthiazol‐2‐yl)‐5‐(3‐carboxymethoxyphenyl)‐2‐(4‐sulfophenyl)‐2*H*‐tetrazolium (MTS) into a colored, soluble formazan whose absorbance at 490 nm is directly proportional to the number of viable cells [46]. Cells were seeded at 5000 cells per well in a 96‐well tissue culture‐treated microplate and incubated for 24 h. Cells were then treated with either QBI‐139 or dmQBI‐139. Samples were serially diluted twofold in PBS without Ca^2+^/Mg^2+^, starting at 30 μM for QBI‐139 and 60 μM for dmQBI‐139. Each treatment volume did not exceed 10% of the medium volume. After 48 h of treatment, 20 μL of MTS reagent was added to the cells. After incubation for ≥2 h at 37°C, the absorbance was recorded at 490 nm. Values represent data collected in biological triplicates with at least three technical replicates and are normalized to the average background absorbance signal from formazan in the medium alone and from vehicle‐treated cells (<10% PBS). Values of IC_50_, which are the concentrations of a ribonuclease that give half‐maximal cell viability, were calculated by fitting the data using a four‐parameter variable slope model (log[agonist] versus response). The significance of QBI‐139 and dmQBI‐139 additions was determined with an unpaired, two‐tailed *t*‐test.

### 2.13 Peptide Synthesis

MVSGWRLFKKIS (HiBiT) and N_3_‐(CH_2_)_5_C(O)‐SGSGVSGWRLFKKIS (azidoHiBiT) were synthesized on a 0.05‐mmol scale on a Rink Amide ProTide resin from CEM (0.59 mmol per gram) and a cycle of 2‐min coupling (90°C), 1‐min deprotection (90°C), and 1‐min associated washes and liquid handling. For azidoHiBiT, the final step was an amide coupling between the N‐terminal serine residue and 6‐azidohexanoic acid from Sigma‐Aldrich. Deprotection was performed with 4‐methylpiperidine (20% v/v) and 1‐hydroxybenzotriazole monohydrate (0.10 M) in DMF. Coupling reactions were performed with 5 equiv. of an Fmoc‐protected amino acid, DIC, and Oxyma in DMF. Peptides were cleaved with 82.5:5:5:2.5:5 TFA/phenol/thioanisole/DODT/H_2_O and purified by HPLC. Pure fractions were combined and lyophilized to obtain a white powder.

### 2.14 Thiol–Maleimide Conjugation

NTB‐capped C4R/P19C/C118V QBI‐139 was subjected to reductive methylation as described in Section 2.6. Cys19 in the unmodified and dimethylated protein was deprotected by the addition of TCEP to a concentration of 5 mM and incubation at 4°C for 15 min. The proteins were exchanged into 50 mM sodium phosphate buffer, pH 7.0, containing EDTA (2 mM) using a Zeba spin desalting column and then incubated for 1 h with a fivefold molar excess of dibenzocyclooctyne‐PEG4‐maleimide, which was from Sigma‐Aldrich (product #760676). Excess maleimide was removed by passing the reaction mixture through a Zeba spin desalting column, exchanging into DPBS without Ca^2+^/Mg^2+^. The final protein concentration was determined by measuring the UV absorption at 280 nm.

### 2.15 Semisynthesis of HiBiT Conjugates

A 20 mM solution of azidoHiBiT was prepared in water. An aliquot of this solution (2 equiv) was added to dibenzocyclooctyne‐labeled dmQBI‐139 and QBI‐139 (1 equiv) in DPBS without Ca^2+^/Mg^2+^. The reaction mixture was incubated overnight with slight agitation. Unreacted peptide was separated from the dmQBI‐139–HiBiT and QBI‐139–HiBiT products using a Micro Bio‐Spin P‐6 column from Bio‐Rad (Waltham, MA) (product #732‐6221) and exchanged into DPBS without Ca^2+^/Mg^2+^.

### 2.16 Cellular Uptake and Stability of HiBiT Conjugates

HEK 293 AAV cells, which constitutively produce cytosolic LgBiT, were seeded at 10,000 cells per well in a cell‐culture, flat clear‐bottom, 96‐well white plate (Fisher Scientific, product 07‐000‐167) 24 h before treatment. Cells were then treated with either dmQBI‐139–HiBiT, QBI‐139–HiBiT, or a HiBiT peptide. After 24 h, the medium was removed, and the cells were washed (2×) with DPBS without Ca^2+^/Mg^2+^. Then, 100 μL of FluoroBrite DMEM (Thermo Fisher Scientific, product #A1896701) containing FBS (10% v/v) and penicillin–streptomycin solution (1% v/v) was added to each well. Luminescence detection was performed using a Nano‐Glo live cell assay kit according to the manufacturer's instructions (Promega, Madison, WI, USA). Luminescence was measured with a Tecan Spark multimode microplate reader. RLU was quantified at 30 min to represent uptake of the variants at 24 h across two concentrations. The signal from cells plus Furimazine substrate was subtracted as background from the dmQBI‐139–HiBiT, QBI‐139–HiBiT, and HiBiT peptide conditions.

HEK 293 AAV cells constitutively producing cytosolic LgBiT were seeded at 10,000 cells per well in a cell‐culture flat clear‐bottom 96‐well white plate (Thermo Fisher Scientific, product #07‐000‐167). After 24 h, cells were treated with 100 nM dmQBI‐139–HiBiT, QBI‐139–HiBiT, or control HiBiT peptide for 14 h. This concentration was the minimum yielding a detectable signal after 12 h in our system. After 14 h, the medium was removed, and cells were washed (2×) with DPBS without Ca^2+^/Mg^2+^. Then, 100 μL of FluoroBrite DMEM (Thermo Fisher Scientific product #A1896701) containing FBS (10% v/v) and penicillin–streptomycin solution (1% v/v) was added to each well. Nano‐Glo Endurazine live‐cell substrate (Promega, Madison, WI, USA) was added to the fresh medium according to the manufacturer's instructions. Luminescence was tracked with a Tecan Spark multimode microplate reader pre‐warmed to 37°C. Cells were maintained in a cell incubator at 37°C, humidified with CO_2_ (5% v/v), and transferred to the plate reader for time‐point analysis. The time points were 2, 4, 6, 8, 14, 24, 34, 48, and 58 h after substrate addition, corresponding to 16, 18, 20, 24, 26, 38, 48, 62, and 72 h of incubation with dmQBI‐139–HiBiT, QBI‐139–HiBiT, or HiBiT. The signal from cells plus Endurazine substrate was subtracted from the signals from dmQBI‐139–HiBiT, QBI‐139–HiBiT, and HiBiT treatments. Each time point represents four replicates per condition.

## 3 Results and Discussion

### 3.1 Strategy for Lysine Modification

As a reliable strategy for chemical lysine modification, we sought a method that is gentle, rapid, and selective while adding minimal mass to the protein. We turned our attention to reductive methylation, which adds only six atoms to an amino group (Figure 1). We prioritized reagents that accommodate mild labeling conditions, unlike sodium cyanoborohydride, which requires harsh reaction conditions and often yields low conversion rates [47]. We used a reductive methylation procedure adapted from protein crystallization methods that, to our knowledge, has not been applied beyond structural analysis [15]. Our reaction conditions tolerated 1–2 mg mL^−1^ of protein, were performed in 50 mM sodium phosphate buffer, pH 7.5, at 4°C, and were completed in <6 h.

**FIGURE 2:**
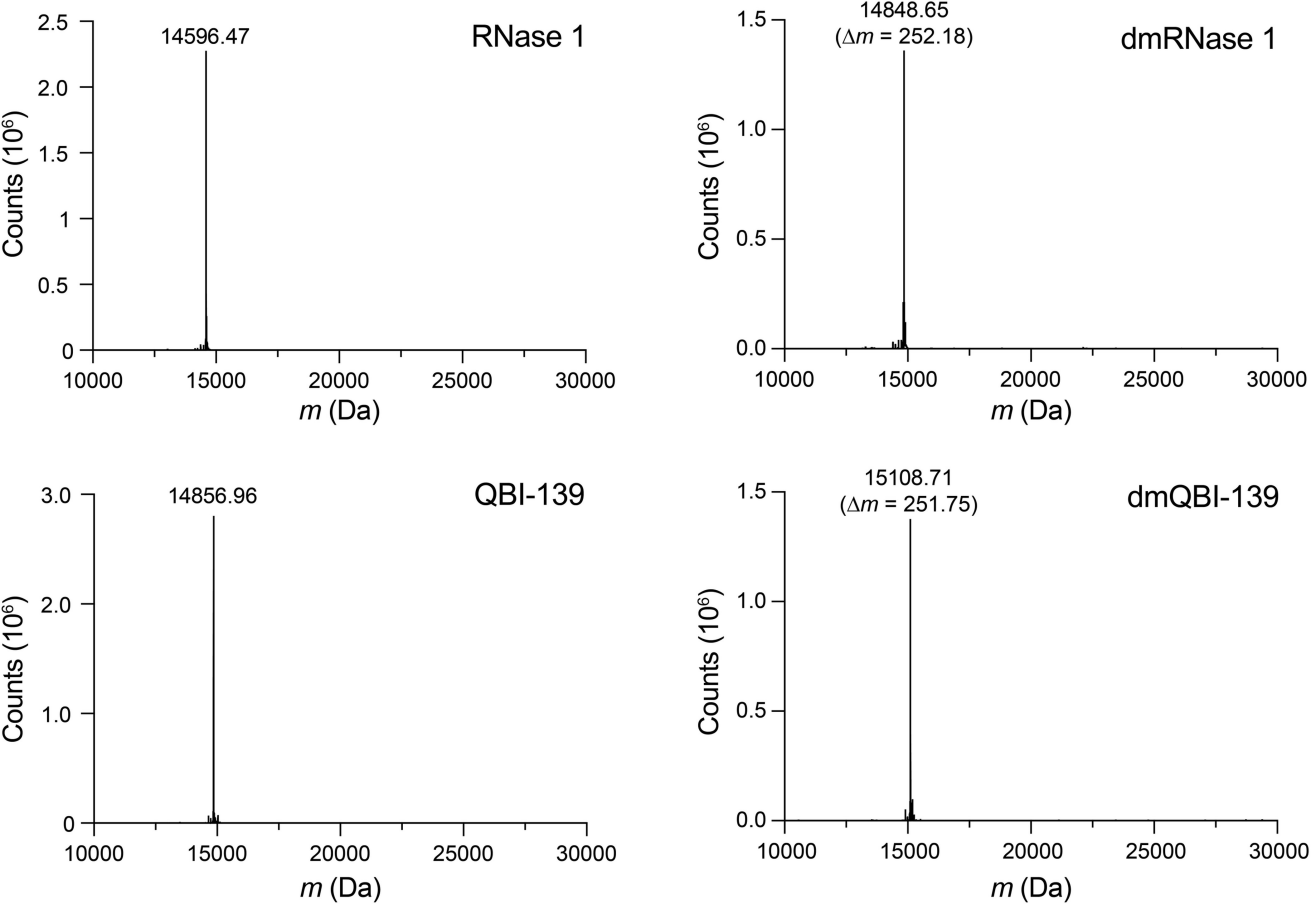
Q‐TOF mass spectra before and after reductive methylation. Each protein has 9 amino groups from 8 lysine residues and an N terminus. *N*,*N*‐Dimethylation yields an expected ∆*m* of 252.49 Da.

Both RNase 1 and QBI‐139 have eight lysine residues and an N terminus; thus, complete labeling should result in a +252 Da shift. In our hands, we observed complete labeling of both RNase 1 and QBI‐139 upon analysis by Q‐TOF mass spectrometry (Figure 2). Hereinafter, we will refer to dimethylated RNase 1 and dimethylated QBI‐139 as dmRNase 1 and dmQBI‐139, respectively.

### 3.2 Effect of Reductive Methylation on Thermostability

**FIGURE 3:**
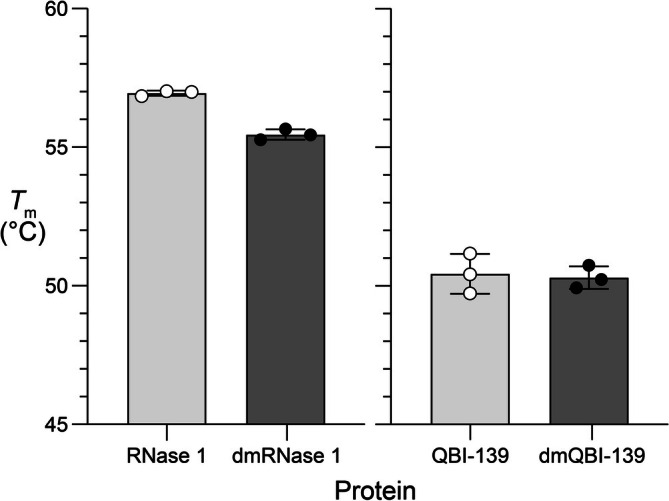
Effect of reductive methylation on thermostability. Values of *T*_m_ (mean ± SD) were determined by differential scanning fluorimetry in 100 mM Tris–HCl buffer, pH 7.5, containing NaCl (100 mM). RNase 1, 57.0°C ± 0.1°C; dmRNase 1, 55.5°C ± 0.2°C; QBI‐139, 50.4°C ± 0.7°C; and dmQBI‐139, 50.3°C ± 0.4°C.

To confirm this strategy as a viable option for biological applications, we first sought to characterize any destabilizing effects on thermostability from the newly introduced methyl groups. We expected that the strategy would not affect *T*_m_ because lysine residues are generally exposed to solvent in folded proteins [48]. Indeed, we observed a shift of ≤1.5°C in *T*_m_ between the unmodified and dimethylated proteins (Figure 3).

### 3.3 Effect of Reductive Methylation on a Protein–Protein Interaction

**FIGURE 4:**
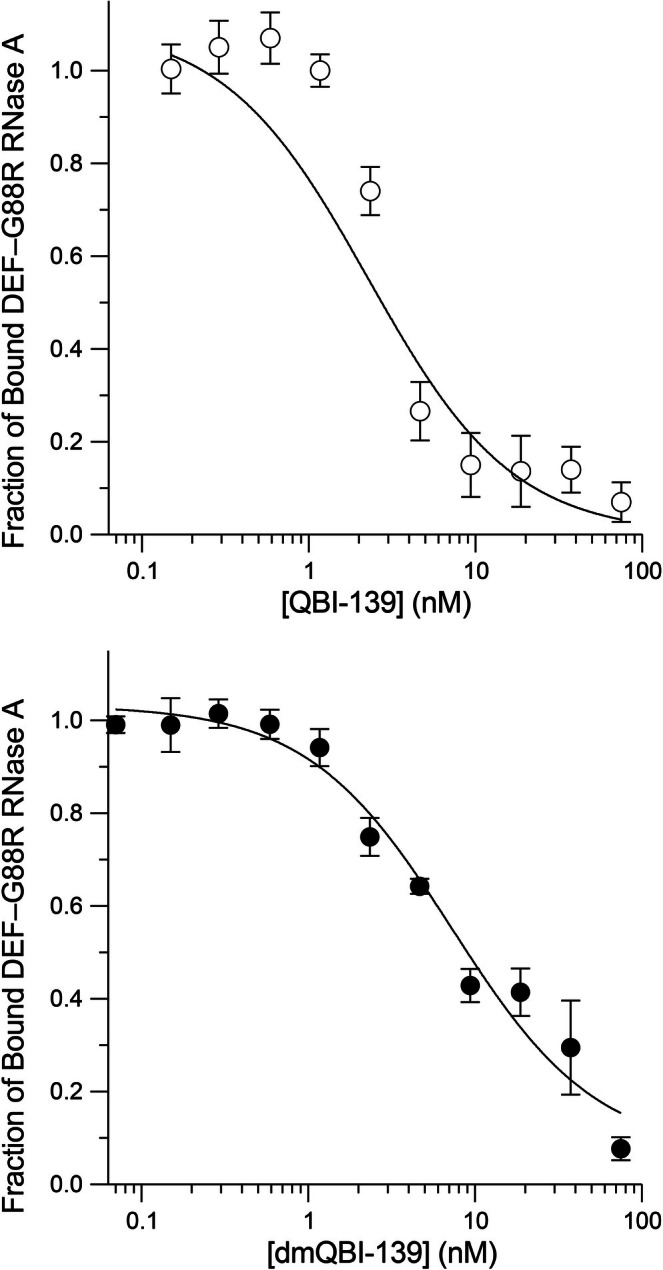
Effect of reductive methylation on a protein–protein interaction. The affinity of QBI‐139 and dmQBI‐139 for RI was measured with a competition assay in PBS containing BSA (0.1 mg mL^−1^) and DTT (10 mM). Values of *K*_d_ (mean ± SD, *n* = 3) are QBI‐139, 0.64 ± 0.08 nM, and dmQBI‐139, 1.8 ± 0.3 nM.

RNase 1 binds to its cognate inhibitor protein, RI, with femtomolar affinity [49]. In contrast, QBI‐139 has an affinity in the high picomolar/low nanomolar range due to engineered steric and electrostatic interactions [38, 50]. For experimental ease, we opted to use QBI‐139 as the model for binding to RI using an assay that exploits a pH‐sensitive fluorescein conjugate whose fluorescence increases upon release from the anionic surface of RI (p*I* 4.7 [51]) into bulk solution [41]. We found that dmQBI‐139 had a *K*_d_ of 1.8 ± 0.3 nM, compared with 0.64 ± 0.08 nM for unmodified QBI‐139 (Figure 4), which is indistinguishable from the *K*_d_ value reported previously for the RI·QBI‐139 complex [37, 38]. Thus, adding 18 CH_2_ groups has only a modest effect on a protein–protein interaction. This tolerance likely reflects the inherent flexibility of interfacial lysine residues, which can reorganize to accommodate the additional steric bulk while maintaining their long‐range electrostatic contributions. Conversely, reductive methylation could serve as a chemical probe to identify specific interfaces where a critical lysine residue is deeply buried or strictly constrained, because inserting methyl groups there would disrupt binding.

### 3.4 Effect of Reductive Methylation on Protein Function

RNase 1 relies primarily on three residues to catalyze the cleavage of a P–O^5′′^ bond in RNA: His12, Lys41, and His119 [40]. Among these residues, a hydrogen bond from Lys41 is key to stabilizing the transition state [16]. In addition, RNase 1 relies on favorable interactions between phosphoryl groups and two other conserved lysine residues: Lys7 and Lys66 [17, 39, 40]. Using 6‐FAM–dArU(dA)_2_–6‐TAMRA, which is a fluorogenic substrate [52], we determined that RNase 1 and dmRNase 1 have *k*_cat_/*K*_M_ values of (3.10 ± 0.19) × 10^6^ and (1.62 ± 0.03) × 10^4^ M^−1^ s^−1^, respectively. Thus, RNase 1 retains only 0.14% of its catalytic activity upon dimethylation. For comparison, replacement of Lys41 in RNase 1 with arginine reduced catalytic activity to 1.6% [53]. The effect of dimethylation on catalysis by QBI‐139 is even more severe. The *k*_cat_/*K*_M_ decreased from (1.73 ± 0.03) × 10^5^ M to (6.0 ± 0.2) × 10^1^ M^−1^ s^−1^, a retention of 0.035%.

**FIGURE 5:**
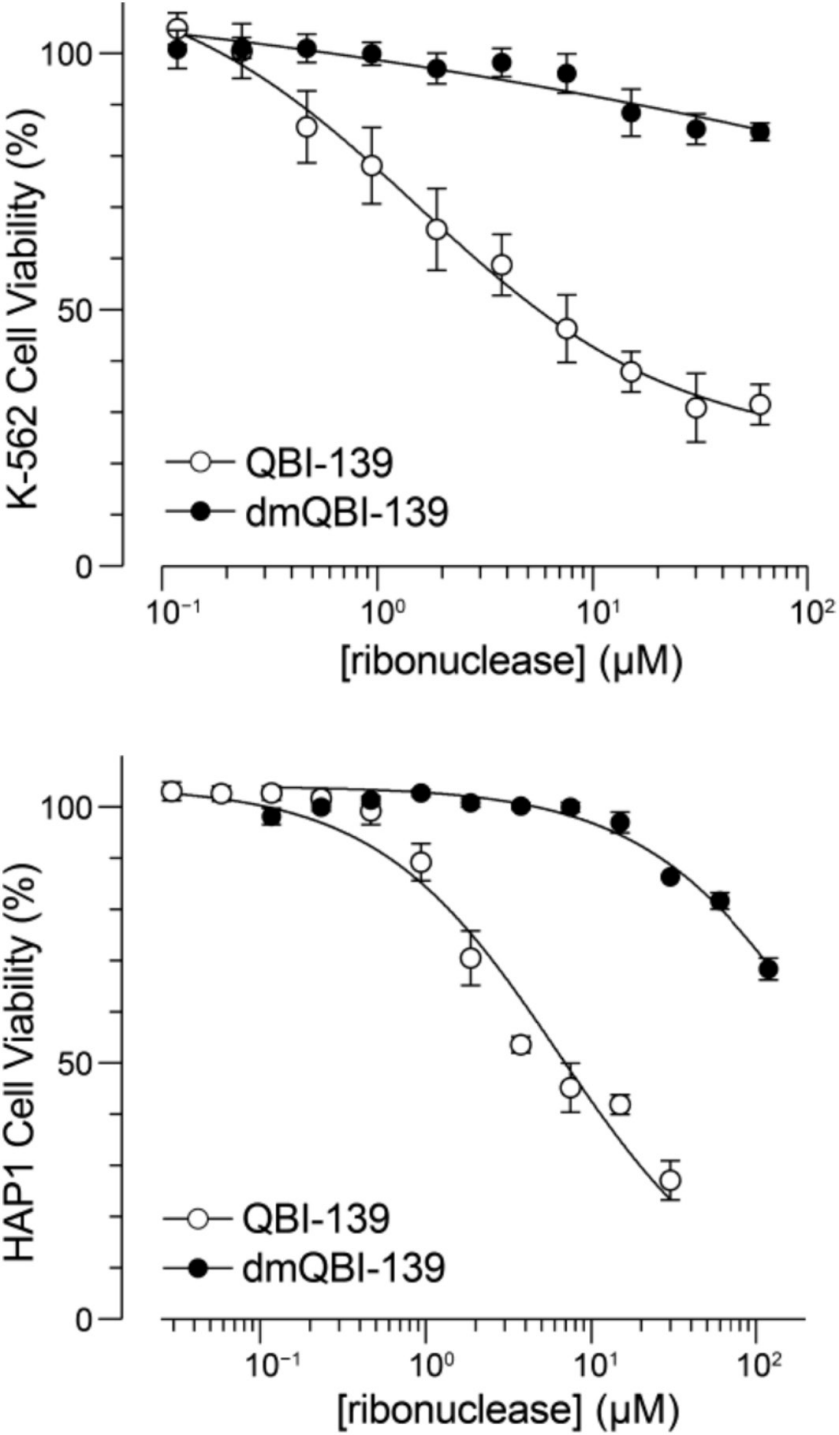
Effect of reductive methylation on the toxicity of ribonucleases for two human cell lines. Values of IC_50_ (mean ± SD, *n* = 3) are K‐562: QBI‐139, 2.3 ± 0.2 μM; dmQBI‐139, >100 μM; HAP1: QBI‐139, 6.5 ± 1.3 μM; and dmQBI‐139, 301 ± 43 μM.

QBI‐139 is known to have enhanced cell‐entry abilities and a weaker association with RI than wild‐type RNase 1 [37, 38]. To assess cytotoxicity, we opted to use K‐562 cells, as they are highly sensitive to ribonucleases [54]. We found the IC_50_ of QBI‐139 to be 2.3 ± 0.2 μM, whereas dmQBI‐139 was not cytotoxic across the tested concentration range (Figure 5). We also assessed toxicity in another leukemia cell line, HAP1, which is nearly haploid and produces fewer copies of proteins than a diploid cell, potentially reducing compensatory effects. In HAP1 cells, we did observe toxicity for dmQBI‐139. The IC_50_ values for the unmodified and dimethylated proteins were 6.5 ± 1.3 and 301 ± 43 μM, respectively. The higher IC_50_ value of dmQBI‐139 is consistent with its diminished ribonucleolytic activity, which is the basis for cytotoxicity [55].

### 3.5 Effect of Reductive Methylation on Cellular Uptake and Stability

**FIGURE 6:**
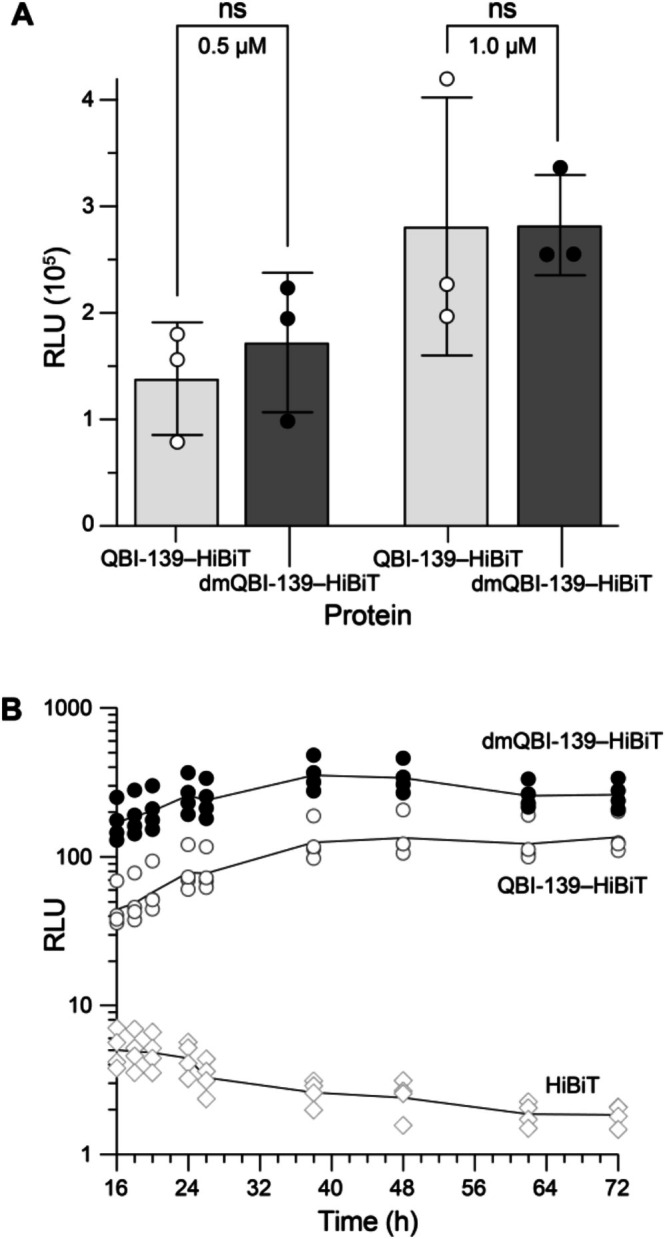
Effect of reductive methylation on cellular uptake and stability using protein–HiBiT conjugates and HEK 293 AAV LgBiT cells. (A) Luminescence observed after 24 h of incubation from two protein concentrations. Values are the mean ± SD (*n* = 3). (B) Luminescence observed over time after incubation with protein–HiBiT conjugates or HiBiT peptide (100 nM). The lines trace the mean value (*n* = 4).

To test for differences in cellular uptake and stability, we implemented a split luciferase assay [56, 57]. The HiBiT fragment contains two lysine residues that would be modified by our reductive methylation protocol if fused genetically. Hence, we developed a semisynthetic strategy to conjugate HiBiT to QBI‐139 *after* reductive methylation. We engineered a variant of QBI‐139 to incorporate a cysteine at residue 19, a modification that does not affect catalysis or RI‐affinity [41, 50, 58, 59]. After reductive methylation, we *S*‐alkylated Cys19 with DBCO‐PEG4‐maleimide and conjugated the DBCO moiety to azido‐HiBiT via strain‐promoted azide–alkyne cycloaddition. The resulting dmQBI‐139–HiBiT conjugate and its unmodified analog were then used to treat human embryonic kidney (HEK) 293 AAV cells that constitutively produce cytosolic LgBiT [60]. We observed no significant difference in uptake at 24 h (Figure 6A); however, during the half‐life tracking studies, we observed a slight initial increase in uptake for dmQBI‐139–HiBiT, perhaps due to its greater hydrophobicity (Figure 1) [61].

Both unmodified and dimethylated QBI‐139–HiBiT persisted in cells with little decay over 72 h (Figure 6B). This persistence is likely attributable to the high intrinsic stability of ribonucleases, given that our control HiBiT treatment showed a detectable loss of signal over time. The higher level of dmQBI‐139–HiBiT compared with QBI‐139–HiBiT persisted throughout the time course.

The orthogonality of an engineered cysteine within a dimethylated scaffold presents an opportunity for further protein and peptide engineering. Following the global reductive methylation of all native amino groups, a unique cysteine residue could be modified with a reagent such as 2‐bromoethylamine. This “cysteine elaboration” yields *S*‐(2‐aminoethyl)cysteine (*γ*‐thialysine), installing a lysine isostere at a user‐defined position [16, 62]. Such a strategy would provide an amino group for site‐specific bioconjugation or ubiquitination on an otherwise fully protected scaffold.

## 4 Conclusions

Reductive methylation converts every primary amino group in a protein to a tertiary dimethylamino group under mild aqueous conditions within 6 h, adding only 6 atoms per site without affecting other functionality. Using RNase 1 and a cytotoxic variant, QBI‐139, we characterized this modification as a physicochemically conservative alternative to the standard genetic strategy of replacing lysine residues with arginine [15].

Dimethylation preserved thermostability (Δ*T*_m_ ≤ 1.5°C) and had only a modest effect on a protein–protein interaction, increasing the *K*_d_ for the RI complex by 3‐fold. Catalytic activity, in contrast, was diminished by 10^2^‐ to 10^3^‐fold, consistent with the steric sensitivity of the ribonuclease active site. This pattern—preservation of global properties, disruption of active‐site function—is informative: it suggests that reductive methylation will be well tolerated in applications that depend on protein stability and binding, such as bioPROTACs, in which efficacy depends on target recruitment rather than enzymatic catalysis. The modification was fully compatible with downstream thiol–maleimide and strain‐promoted azide–alkyne cycloaddition chemistry, supporting its integration into multistep bioconjugation workflows.

Unlike site‐directed mutagenesis, reductive methylation also modifies the N‐terminal *α*‐amino group, which is itself a substrate for ubiquitination [11, 30]. A lysine‐free protein with an acetylated N terminus has been shown to have an increased half‐life [13], but genetic approaches alone cannot modify this site. Reductive methylation addresses that limitation.

Notably, both unmodified and dimethylated ribonucleases showed no measurable decay in signal over 72 h in a live‐cell luminescence assay, indicating that the intrinsically stable ribonuclease fold resists intracellular degradation regardless of methylation status. A protein with lower intrinsic stability would likely be required to resolve kinetic differences between unmodified and dimethylated lysine residues and could be the subject of further study. As tools for intracellular protein delivery continue to advance, reductive methylation offers a practical, generalizable chemical strategy to enhance the intracellular performance of protein‐based therapeutics, including bioPROTACs.

## Funding

C.S.G. was supported by a National Defense Science and Engineering Graduate Fellowship sponsored by the US Air Force Research Laboratory. E.C.W. was supported by a Graduate Research Fellowship from the US National Science Foundation. This work was supported by the US National Institutes of Health (R35 GM148220 and P30 CA014051).

## Conflicts of Interest

The authors declare no conflicts of interest.

## Supporting information


**Table S1:** Abbreviations used.
**Table S2:** Mass spectrometry of proteins and peptides.
**Figure S1:** Analytical HPLC chromatograms of synthetic peptides.

## Data Availability

The data that support the findings of this study are available in the Supporting Information of this article.
