# Supplementary material for: Reductive Methylation: An Alternative to Lysine → Arginine Mutagenesis
Source: J Pept Sci. 2026 Jun 16;32(7):e70110. doi: 10.1002/psc.70110 (PMC13270353; doi:10.1002/psc.70110)
Supplement: Supplementary file 1 — Table S1: Abbreviations used. Table S2: Mass spectrometry of proteins and peptides. Figure S1: Analytical HPLC chromatograms of synthetic peptides. [file PSC-32-e70110-s001.pdf]

Reductive methylation: An alternative to lysine→arginine mutagenesis

Oscar J. Molina[1] | Clair S. Gutierrez[2] | Jinyi Yang[2] | Evans C. Wralstad[2] | Ronald T. Raines[2]

[1]Department of Biology, Massachusetts Institute of Technology, Cambridge, Massachusetts, USA | [2]Department of Chemistry, Massachusetts Institute of Technology, Cambridge, Massachusetts, USA

Correspondence: Ronald T. Raines (rtraines@mit.edu)

Table of Contents

Table of Contents S1
Table S1 | Abbreviations Used S2
Table S2 | Mass Spectrometry of Proteins and Peptides S4
Figure S1 | Analytical HPLC chromatograms of synthetic peptides. S4

Table S1 | Abbreviations Used

**Abbreviation**	**Definition**
ACN	acetonitrile
BCA	bicinchoninic acid
BSA	bovine serum albumin
cLog*P*	calculated logarithm of the *n*-octanol/water partition coefficient
DBCO	dibenzocyclooctyne
DEF	2′,7′-diethylfluorescein
DEFIA	2′,7′-diethylfluorescein-5-iodoacetamide
DIC	*N*,*N*′-diisopropylcarbodiimide
DMEM	Dulbecco’s modified Eagle’s medium
DMF	*N*,*N*-dimethylformamide
DODT	2,2′-(ethylenedioxy)diethanethiol
DPBS	Dulbecco’s phosphate-buffered saline
DTNB	5,5′-dithiobis(2-nitrobenzoic acid)
DTT	dithiothreitol
EDTA	ethylenediaminetetraacetic acid
ESI	electrospray ionization
6-FAM	6-carboxyfluorescein
FBS	fetal bovine serum
Fmoc	fluorenylmethyloxycarbonyl
FPLC	fast protein liquid chromatography
HEK	human embryonic kidney
HiBiT	high-affinity NanoBiT peptide tag
HPLC	high-performance liquid chromatography
IC_{50}	half-maximal inhibitory concentration
IMDM	Iscove’s modified Dulbecco's medium
IPTG	isopropyl-β-D-1-thiogalactopyranoside
K_d	equilibrium dissociation constant
k_{cat}/K_M	specificity constant
LC/MS	liquid chromatography/mass spectrometry
LgBiT	large NanoBiT subunit
MALDI-TOF	matrix-assisted laser desorption/ionization–time of flight
MTS	3-(4,5-dimethylthiazol-2-yl)-5-(3-carboxymethoxyphenyl)-2-(4-sulfophenyl)-2*H*-tetrazolium
MWCO	molecular weight cutoff
NEDD8	neural precursor cell expressed developmentally downregulated protein 8
NTB	2-nitro-5-thiobenzoate

Abbreviation	Definition
OD	optical density
Oxyma	ethyl cyanohydroxyiminoacetate
PBS	phosphate-buffered saline
PEG	polyethylene glycol
PROTAC	proteolysis-targeting chimera
bioPROTAC	biological proteolysis-targeting chimera
Q-TOF	quadrupole time-of-flight
RI	ribonuclease inhibitor
RLU	relative luminescence units
RNase 1	ribonuclease 1 (EC 4.6.1.18; *Homo sapiens*: UniProtKB P07998)
RNase A	ribonuclease A (EC 4.6.1.18; *Bos taurus*: UniProtKB P61823)
RPMI	Roswell Park Memorial Institute
SD	standard deviation
SDS–PAGE	sodium dodecyl sulfate–polyacrylamide gel electrophoresis
SE	standard error
SUMO	small ubiquitin-like modifier
6-TAMRA	6-carboxytetramethylrhodamine
TB	terrific broth
TCEP	tris(2-carboxyethyl)phosphine
TFA	trifluoroacetic acid
T_m	temperature at the midpoint of thermal denaturation

Table S2 | Mass Spectrometry of Proteins and Peptides

	m, calc'd (Da)	*m*, obs'd (Da)	Δ*m* (Da)	Comment
**Unmodified Proteins**				
RNase 1	14,566.24	14,596.47	+30.23	2 Met(O)
QBI-139	14,856.72	14,856.96	+0.24	
A19C/G88R RNase A	13,944.59	13,944.66	+0.07	
ribonuclease inhibitor	49,823.54	49,843.33	+19.79	1 Met(O)
**Modified Proteins**				
dmQBI-139	15,108.72	15,108.71	−0.01	
dmRNase 1	14,818.20	14,848.65	+30.45	2 Met(O)
C4R/P19C(NTB)/C118V QBI-139	15110.99	15111.29	+0.30	
A19C(NTB)/G88R RNase A	14,142.59	14,141.81	−0.78	
DEF–G88R RNase A	14,389.59	14,388.21	−1.38	
QBI-139–DBCO	15,588.59	15,588.76	+0.17	
QBI-139–HiBiT	17,335.54	17,336.76	+1.22	
dmQBI-139–DBCO	15,840.59	15,840.55	−0.04	
dmQBI-139–HiBiT	17,587.54	17,588.26	+0.72	
**Peptides**				
HiBiT	1,450.81	1,452.95	+2.15	
azidoHiBiT	1,746.95	1,749.10	+2.15	

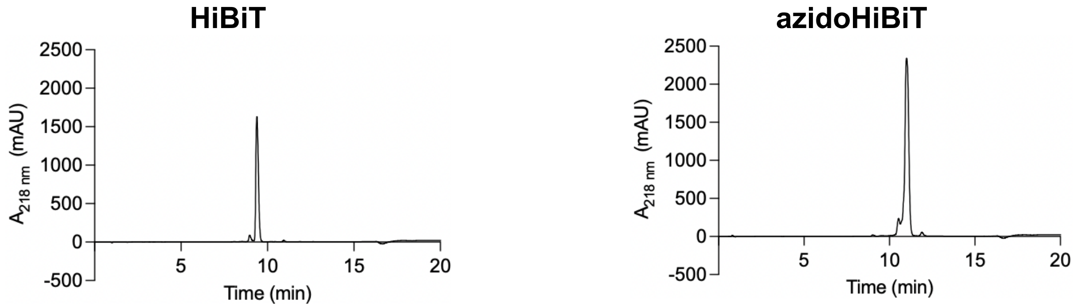

Figure S1 | Analytical HPLC chromatograms of synthetic peptides. Method: 0–40% v/v B over 15 min with A: H_2O/ACN/TFA 95/5/0.1; B: ACN/H_2O/TFA 95/5/0.1.
